# Relational emotional neglect in contexts of abundance: a developmental, relational, and psychoanalytic perspective

**DOI:** 10.3389/fpsyg.2026.1823367

**Published:** 2026-10-22

**Authors:** Renata Nicurgo

**Affiliations:** Independent Researcher and Private Practice, Uberlândia, Minas Gerais, Brazil

**Keywords:** attachment, child development, developmental trauma, emotional availability, emotional neglect, relational emotional neglect

## Abstract

This theoretical article proposes the construct of Relational Emotional Neglect in Contexts of Abundance (RENCA) to describe a relational configuration in which high levels of material provision, educational investment, and functional caregiving coexist with comparatively limited opportunities for emotional attunement, co-regulation, and sustained relational availability. Although emotional neglect has long been recognized within developmental and clinical literature, less attention has been devoted to situations in which emotional deprivation may remain difficult to identify because it unfolds within highly organized and socially privileged caregiving environments. Drawing on Winnicottian psychoanalysis, attachment theory, and contemporary literature on developmental trauma, this perspective article examines how persistent discrepancies between functional caregiving and relational-emotional availability may influence emotional development. Rather than proposing a diagnostic category, RENCA is presented as a theoretical framework intended to organize existing theoretical perspectives, generate hypotheses for future empirical investigation, and promote greater clinical sensitivity to subtle forms of emotional deprivation that may remain socially invisible despite extensive material and developmental investment.

## Introduction

Emotional neglect is widely recognized as a significant developmental risk factor and has been extensively discussed within attachment theory, psychoanalysis, and developmental psychology. Unlike more overt forms of maltreatment, emotional neglect is typically characterized by persistent failures in emotional responsiveness, attunement, validation, and co-regulation, compromising the child’s opportunity to experience consistent relational security and emotional recognition. These failures may occur across diverse family and socioeconomic contexts and are not limited to situations of material deprivation.

Contemporary attachment research indicates that children’s emotional security depends more on responsive relational experience than on material provision or time alone. A recent meta-analysis of 174 studies and 22,914 parent–child dyads found a positive association between caregiver sensitivity and attachment security (*r* = 0.25), with comparable contributions from maternal and paternal sensitivity (Madigan et al., 2024). Secure attachment therefore emerges through repeated experiences of sensitive, predictable, and emotionally attuned care (Bowlby, 1969, 1988; Ainsworth et al., 1978; Duschinsky, 2020). Developmental trauma research likewise associates chronic disruptions in emotional availability with difficulties in affect regulation, self-organization, and interpersonal functioning (Schore, 2001; Teicher and Samson, 2016).

However, one relational configuration has received comparatively limited conceptual attention. In some family environments, children experience high levels of material provision, educational investment, structured routines, extracurricular opportunities, and functional caregiving while simultaneously encountering comparatively limited opportunities for emotional attunement and sustained relational availability. In these situations, caregivers may be consistently present in the practical organization of the child’s life while remaining less available to recognize, contain, and elaborate the child’s subjective emotional experience.

This article does not suggest that emotional neglect is unique to affluent families or that abundance is itself a developmental risk. Rather, it examines a possible discrepancy between high functional provision and limited relational-emotional availability.

To describe this specific configuration, this article proposes the construct of Relational Emotional Neglect in Contexts of Abundance (RENCA). Within this framework, abundance refers not exclusively to financial wealth but more broadly to environments characterized by extensive material resources, educational opportunities, organized caregiving, and high levels of developmental investment. The defining feature of RENCA is therefore not socioeconomic status itself, but the discrepancy between functional caregiving and emotional availability.

RENCA is a provisional theoretical construct, not a diagnostic category or validated syndrome. It is intended to integrate established perspectives, clarify a potentially meaningful relational pattern, and generate testable hypotheses.

## Theoretical framework

### Attachment theory and emotional availability

Attachment theory provides one of the most robust developmental frameworks for understanding how early relational experiences influence emotional development. Bowlby (1969, 1988) proposed that children develop internal working models of themselves and others through repeated interactions with caregivers, particularly during situations involving distress, uncertainty, and the need for protection. These internal representations contribute to later expectations regarding emotional availability, trust, and interpersonal relationships throughout development.

Building upon Bowlby’s theoretical model, Ainsworth et al. (1978) demonstrated empirically that caregiver sensitivity plays a central role in the development of attachment security. Sensitive caregivers consistently recognize, interpret, and respond appropriately to children’s emotional signals, fostering a sense of safety that supports emotional regulation, exploration, and psychological development.

Schaffer and Emerson’s (1964) longitudinal observations suggested that attachment formation was more closely associated with caregiver responsiveness than with the amount of physical care or time spent together. Contemporary meta-analytic evidence supports this emphasis: caregiver sensitivity is positively associated with attachment security across maternal and paternal relationships (Madigan et al., 2024). Attachment is therefore best understood as a dynamic process shaped by repeated emotionally responsive interactions across changing contexts (Duschinsky, 2020).

From this perspective, emotional availability cannot be reduced to physical presence, supervision, or functional caregiving. Caregivers may successfully organize routines, provide educational opportunities, ensure physical safety, and meet developmental milestones while differing considerably in their capacity to recognize, contain, validate, and co-regulate children’s emotional experiences. This distinction between functional caregiving and emotional responsiveness provides an important conceptual foundation for the present proposal.

### Winnicottian psychoanalysis and the development of the self

A complementary understanding emerges from Winnicott’s theory of emotional development. Winnicott (1965) described healthy development as depending upon a facilitating environment capable of providing holding, continuity, and sufficiently responsive caregiving during early development. Through repeated experiences of being emotionally recognized and psychologically held, children gradually develop spontaneity, emotional integration, and a stable sense of self.

Conversely, when caregivers consistently fail to acknowledge or sustain the child’s subjective emotional experience, adaptation may increasingly occur through compliance with external expectations. Winnicott described this developmental process through the concept of the false self, whereby externally successful adaptation may coexist with diminished spontaneity and reduced access to authentic emotional experience.

Importantly, Winnicott did not conceptualize these processes as deterministic outcomes resulting from isolated caregiving failures. Rather, emotional development reflects cumulative relational experiences occurring across time, emphasizing the importance of consistent emotional responsiveness rather than isolated interactions.

### Developmental trauma and mentalization

Contemporary developmental trauma literature further supports the importance of emotional responsiveness for psychological development. Chronic failures in emotional attunement have been associated with difficulties in affect regulation, stress responsivity, interpersonal functioning, and self-organization (Schore, 2001; Teicher and Samson, 2016). These associations are understood as probabilistic developmental pathways influenced by multiple biological, relational, and environmental factors rather than as inevitable consequences of specific caregiving experiences.

Similarly, mentalization theory proposes that children’s capacity to understand both their own and others’ mental states develops through relationships in which emotions are consistently reflected, named, and experienced as meaningful (Fonagy et al., 2002; Fonagy and Allison, 2014). Emotional development therefore depends not solely upon protection or material provision but also upon repeated opportunities to experience psychological recognition within close relationships.

Taken together, attachment theory, Winnicottian psychoanalysis, developmental trauma research, and mentalization theory converge in emphasizing that children’s development depends fundamentally upon emotionally responsive relationships. These complementary perspectives provide the theoretical basis for examining relational configurations in which extensive functional caregiving may coexist with comparatively limited emotional availability.

## Conceptual specificity of RENCA

Emotional neglect is a well-established construct within developmental and clinical literature and has been described across diverse family and socioeconomic contexts. The present article does not propose a new form of neglect, nor does it suggest that emotional neglect is exclusive to affluent families or environments characterized by material abundance.

Rather, RENCA is proposed as a theoretical construct intended to describe a specific relational configuration in which substantial functional investment coexists with comparatively limited emotional availability. Its defining feature is therefore neither wealth nor privilege themselves, but the discrepancy between the quality of functional caregiving and the quality of relational-emotional engagement.

Within this framework, abundance should be understood broadly. It encompasses not only financial resources but also extensive educational opportunities, highly structured routines, extracurricular activities, technological resources, specialized services, and intensive parental investment in children’s objective development. These resources are not conceptualized as inherently problematic. On the contrary, they may represent important protective factors that promote children’s cognitive, educational, and social development.

The theoretical question proposed by RENCA concerns situations in which these forms of functional investment progressively assume relational functions that cannot be fully replaced by material provision or organized developmental opportunities. In such circumstances, caregivers may successfully provide opportunities, supervision, and practical support while offering comparatively fewer experiences of emotional attunement, reflective dialogue, shared emotional meaning, and co-regulation.

Importantly, RENCA should not be interpreted as suggesting that structured routines, technology, extracurricular activities, or therapeutic interventions produce emotional neglect. These resources can enrich children’s development and strengthen family functioning. The proposed construct instead invites consideration of whether, in some circumstances, they may unintentionally operate as substitutes for sustained relational engagement when emotional availability becomes chronically limited.

This distinction differentiates RENCA from emotional neglect considered more broadly. Traditional descriptions of emotional neglect encompass diverse relational failures across multiple developmental contexts. RENCA, by contrast, focuses specifically on the coexistence of high functional provision and comparatively restricted emotional responsiveness, proposing that this discrepancy may constitute a recognizable relational pattern worthy of theoretical examination.

Accordingly, RENCA is introduced as a provisional conceptual framework rather than as a diagnostic category or empirically validated syndrome. Its purpose is to organize clinical observations, refine theoretical discussion, and generate empirically testable hypotheses regarding developmental processes that may remain relatively invisible when assessments emphasize material provision and functional caregiving alone.

## Provisional clinical-developmental configurations across development

Developmental capacities, social expectations, and emotional tasks shape how relational adversity is expressed over time. The configurations below are therefore stage-specific, provisional hypotheses derived from attachment, mentalization, and Winnicottian theory rather than diagnostic profiles.

They illustrate how a persistent discrepancy between functional caregiving and emotional availability might become visible at different developmental periods and require empirical testing.

### Preschool years

During the preschool years, emotional development relies heavily on co-regulation, symbolic play, emotional naming, and caregiver responsiveness. Meta-analytic evidence from 36 studies involving 3,847 children found that greater caregiver sensitivity was associated with secure and organized preschool attachment (O’Neill et al., 2021). Within RENCA, a child may show age-appropriate cognitive and behavioral competence while having fewer opportunities for emotional elaboration, shared affect, and supported regulation.

A possible adaptation is compliance, precocious autonomy, or behavioral competence rather than overt dysregulation. From a Winnicottian perspective, this may reflect increasing adaptation to external expectations when spontaneity receives less recognition; it remains a hypothesis requiring direct study.

### School age

At school age, children’s relational worlds expand, but caregiver availability remains important for emotional security. Meta-analytic evidence on the Security Scale links attachment security in middle childhood with school adaptation, emotional and peer competence, self-esteem, and fewer behavioral problems (Brumariu et al., 2018). Within RENCA, high academic performance and organized routines may coexist with difficulty identifying, expressing, or sharing emotional experience.

Achievement or structured activities may then become prominent sources of validation. This pattern is not evidence of psychopathology and should be tested as a possible adaptation to limited emotional reciprocity rather than treated as an expected outcome.

### Adolescence

Adolescence intensifies demands for identity formation, emotional autonomy, and interpersonal intimacy. Meta-analytic evidence across 123 samples found a moderate association between insecure attachment and depressive symptoms in children and adolescents (*r* = 0.31), while also showing substantial variation across studies (Spruit et al., 2020). Within RENCA, persistent discrepancies between functional support and emotional availability may therefore be examined in relation to emotional authenticity, help-seeking, and communication of subjective needs.

Some adolescents may maintain high academic, extracurricular, or social functioning while reporting emotional emptiness or relational loneliness. These are non-specific, probabilistic hypotheses and must not be interpreted as characteristic or causal outcomes of RENCA.

### Developmental continuity

Across all developmental stages, the central hypothesis proposed by RENCA remains unchanged. The construct does not assume that material resources, structured routines, or educational opportunities are harmful. Instead, it proposes that developmental outcomes may differ when these important forms of functional caregiving consistently coexist with comparatively limited opportunities for emotional attunement, shared meaning-making, and sustained relational availability.

Across stages, these configurations are heuristic and require longitudinal, culturally sensitive testing.

### Relational substitution in contexts of abundance

One of the central theoretical assumptions of the present framework concerns the possibility that functional caregiving and emotionally responsive caregiving may gradually become dissociated within certain contexts of material abundance. Under these circumstances, children’s developmental needs may be addressed through an increasingly sophisticated network of educational opportunities, extracurricular activities, technological resources, and specialized services, while opportunities for emotionally meaningful interactions become comparatively less frequent. The present framework refers to this process as relational substitution, emphasizing that functional investment and emotional availability represent complementary rather than interchangeable dimensions of caregiving.

Importantly, this proposal does not suggest that educational programs, digital technologies, extracurricular activities, or therapeutic interventions are inherently detrimental to child development. On the contrary, these resources can promote learning, socialization, emotional support, and developmental growth. The theoretical concern arises only when such resources progressively assume relational functions that historically depended upon emotionally attuned interactions between children and their primary caregivers. Within this perspective, developmental opportunities should be understood as enriching relational experiences rather than replacing them.

Contemporary childhood may be highly structured by academic expectations, extracurricular commitments, digital technologies, and performance demands. These experiences can provide substantial developmental advantages, but may also narrow opportunities for reciprocity, shared attention, and spontaneous interaction when family routines become chronically overprogrammed.

The relevant question is not whether stimulation, technology, or treatment is inherently harmful, but whether they enrich or progressively replace emotionally responsive relationships. This may be especially relevant for atypically developing children receiving multiple specialized interventions when intensive therapeutic programming unintentionally restricts spontaneous, emotionally attuned interaction with primary caregivers.

This conceptual perspective is particularly relevant within contemporary family systems in which caregiving responsibilities are increasingly distributed across schools, healthcare professionals, digital platforms, extracurricular programs, and other institutional settings. Although such arrangements often reflect substantial parental investment and genuine concern for children’s development, the proposed model suggests that emotional availability remains a distinct relational function that cannot be fully delegated or substituted through external resources, regardless of their quality or abundance.

Accordingly, relational substitution should be understood not as a characteristic of specific parenting practices, but as a theoretical lens through which contemporary caregiving arrangements may be examined. By emphasizing the complementarity between functional caregiving and emotionally responsive relationships, this perspective extends existing discussions on emotional neglect while remaining fully consistent with attachment theory and broader developmental psychology.

## Future directions for empirical research

Relational Emotional Neglect in Contexts of Abundance (RENCA) is proposed as a theoretical construct intended to integrate existing perspectives on attachment, emotional availability, relational development, and emotional neglect into a coherent conceptual framework. Rather than introducing a new diagnostic category or a definitive explanatory model, the present proposal seeks to generate theoretically grounded hypotheses that may guide future empirical investigation.

An important next step will be to examine whether RENCA represents a distinguishable relational configuration within the broader spectrum of emotional neglect or whether it is more appropriately understood as one manifestation of already established developmental constructs. Addressing this question will require empirical studies capable of evaluating the construct’s conceptual coherence, developmental significance, discriminant validity, and applicability across diverse family and cultural contexts.

Future research may employ multiple methodological approaches to investigate the theoretical propositions advanced in this article. Longitudinal studies could examine developmental trajectories associated with discrepancies between high levels of functional caregiving and comparatively limited emotional availability across childhood and adolescence. Observational research may explore caregiver-child interactions characterized by abundant practical investment alongside reduced emotional attunement, whereas qualitative studies may contribute by examining how adolescents and adults retrospectively describe experiences of growing up in materially supportive but emotionally distant relational environments.

Operationalization could begin with a discrepancy profile combining (a) indicators of high functional investment—material provision, educational opportunities, organized routines, supervision, and access to services—with (b) validated measures of low emotional availability, including caregiver sensitivity, attunement, reflective dialogue, reciprocity, and co-regulation. Multimethod assessment should combine caregiver and child reports, observational coding of caregiver-child interaction, developmentally appropriate attachment measures, and longitudinal follow-up. Researchers could then test whether the magnitude and persistence of the functional-emotional discrepancy predict outcomes beyond emotional neglect, attachment insecurity, socioeconomic status, and general family stress, thereby evaluating reliability, construct validity, incremental validity, and discriminant validity.

Cross-cultural investigations will be particularly important, given that caregiving practices, expressions of emotional responsiveness, and family organization vary considerably across societies. Examining the proposed framework across diverse cultural settings may help clarify whether the relational configuration described by RENCA reflects universal developmental processes or context-specific caregiving patterns.

Accordingly, the present article should be understood as an initial theoretical contribution intended to stimulate empirical investigation rather than as a definitive explanatory model. The long-term scientific contribution of RENCA will ultimately depend on future research capable of refining its conceptual boundaries, evaluating its empirical validity, and determining its contribution to developmental and clinical psychology.

## Discussion

The present article introduced Relational Emotional Neglect in Contexts of Abundance (RENCA) as a theoretical framework intended to describe a relational configuration in which high levels of functional caregiving coexist with comparatively limited emotional availability. Rather than proposing a new form of neglect or a diagnostic category, the proposed framework was developed to integrate established theoretical perspectives and address a conceptual gap concerning children’s relational experiences within contexts characterized by material abundance and substantial functional investment.

The model integrates attachment, emotional availability, developmental trauma, and psychoanalytic perspectives to address situations in which practical and developmental needs are extensively met while emotionally meaningful interaction remains limited.

Its contribution is to shift attention from the quantity of provision to the relational quality of everyday care. Abundance is not a risk factor; the relevant hypothesis is that extensive functional investment may coexist with limited emotional reciprocity.

Relational substitution further emphasizes that educational, technological, extracurricular, and therapeutic resources, although often beneficial or essential, cannot replace emotionally attuned relationships. The hypothesis is especially relevant where caregiving is distributed across institutions or where intensive intervention schedules for atypically developing children may reduce unstructured relational time.

At the same time, several limitations should be acknowledged. The construct remains theoretical and has not yet undergone empirical validation. Consequently, the developmental configurations described throughout this article should be interpreted as theoretically derived hypotheses rather than established developmental outcomes. Likewise, its conceptual boundaries, discriminant validity, and cross-cultural applicability remain open questions requiring systematic empirical investigation.

## Conclusion

RENCA organizes an underexplored configuration within the literature on emotional neglect: high functional caregiving may coexist with limited emotional attunement.

The framework does not portray material abundance as harmful. It emphasizes that practical care and developmental opportunities cannot replace emotionally responsive relationships.

As a proposal for conceptual integration and hypothesis generation, RENCA will depend on future research testing its boundaries, validity, and usefulness across developmental and cultural contexts.

## Data Availability

The original contributions presented in the study are included in the article/supplementary material, further inquiries can be directed to the corresponding author.
